# A First Approach for the Micropropagation of the Edible and Medicinal Halophyte *Inula crithmoides* L.

**DOI:** 10.3390/plants12122366

**Published:** 2023-06-19

**Authors:** Maria João Rodrigues, Viana Castañeda-Loaiza, Eliana Fernandes, Luísa Custódio

**Affiliations:** Centre of Marine Sciences, Faculty of Sciences and Technology, University of Algarve, Campus of Gambelas, 8005-139 Faro, Portugal

**Keywords:** golden samphire, salt-tolerant plants, plant tissue culture, in vitro multiplication

## Abstract

*Inula crithmoides* L. (golden samphire) is an edible aromatic halophyte species with confirmed nutritional and medicinal properties attributed to the presence of important metabolites, including proteins, carotenoids, vitamins, and minerals. Therefore, this study aimed at establishing a micropropagation protocol for golden samphire that can serve as a nursery approach to its standardized commercial cultivation. For that purpose, a complete regeneration protocol was developed by improving shoot multiplication from nodal explants, rooting, and acclimatization methodologies. The treatment with BAP alone induced the maximum shoot formation (7–7.8 shoots/explant), while IAA treatment increased the shoot height (9.26–9.5 cm). Furthermore, the treatment that coupled best shoot multiplication (7.8 shoots/explant) and highest shoot height (7.58 cm) was MS medium supplemented with 0.25 mg/L BAP. Moreover, all shoots produced roots (100% rooting), and multiplication treatments did not exert significant effect on root length (7.8–9.7 cm/plantlet). Moreover, by the end of the rooting phase, plantlets cultivated with 0.25 mg/L BAP had the highest shoot number (4.2 shoots/plantlet), and plantlets from 0.6 mg/L IAA + 1 mg/L BAP presented the highest shoot height (14.2 cm) similar to control plantlets (14.0 cm). The survival up to the ex-vitro acclimatization stage was increased from 9.8% (control) to 83.3%, when plants were treated with a paraffin solution. Nevertheless, the in vitro multiplication of golden samphire is a promising way for its rapid propagation and can be used as a nursery method, contributing to the development of this species as an alternative food and medicinal crop.

## 1. Introduction

Halophytes thrive in saline environments, such as salt marshes, saline arid lands, maritime dunes, and cliffs, and are subjected to severe abiotic conditions (e.g., high salinity, high light intensity, drought, flooding, and severe temperature variations) [1]. The high content of chloride and sodium ions in the soil and water is highly toxic to plant tissues, leading to high-stress levels in plants (e.g., hyperosmotic shock, nutrient imbalance, stomatal closure, inhibition of photosynthesis, and decreased water intake) [2]. To handle these constraints, halophytes developed different physiological and biochemical adaptations, including preservation of osmotic homeostasis, production of antioxidant enzymes, and of antioxidant metabolites [3,4,5,6,7]. Those metabolites confer them important nutritional and functional traits, such as antioxidant, anti-inflammatory, and antitumor properties, and are therefore of high interest for food and pharmaceutical industries [8].

*Inula crithmoides* L. (syn. *Limbarda crithmoides* (L.) Dumort), commonly known as golden samphire, is an aromatic halophyte species belonging to the Asteraceae family, and is scattered along the Mediterranean coastal saltmarshes and saline reedbeds [9]. It is considered as an edible plant since its young leaves or shoots can be eaten raw, cooked, or pickled in vinegar, particularly in Lebanon, Spain, and Italy [10,11], and it has ethnomedicinal uses for treating rheumatic pain and goiter [9,12]. Moreover, scientific studies disclosed that golden samphire is rich in iodine, proteins, carotenoids (β-carotene and lutein), water-soluble vitamins (B1 and B6), and important minerals (sodium, potassium, and iron), and that it exhibits medicinal properties, such as antioxidant [10,13,14] and antibacterial [11]. In addition, it was considered by a panel of culinary experts, including reputed cuisine chefs, to be a very appetizing plant for human consumption due to its high-sensory attributes [14]. Due to its organoleptic and medicinal properties and high salt tolerance, golden samphire can be a candidate in saline agriculture that targets its use as food or to provide bioactive ingredients for pharma or cosmetic sectors.

Increasing soil and freshwater salinization are the main limiting factors in crop production in arid and semiarid regions, leading to increased food insecurity. This leads to the search for innovative, new commercial crops that are more adapted to saline conditions, such as halophytes. In the mid-1990’s, Zurayk and Baalbaki [10] explored the potential of cultivating golden samphire in saline conditions, obtaining successful growth and propagation for both freshwater and saltwater irrigation outside its native environment [10]. The recent attempts by RiaFresh, a local Portuguese company producing edible halophytes for culinary use, have also been successful in growing golden samphire in a soilless system under different levels of salinity [14]. However, this species presented severe restriction of germination above 20 dS/m, which indicates that vegetative multiplication must be a central strategy for saline species [10]. However, despite its commercial potential and cultivation attempts, there are no widespread cultivation methods for the nursery of golden samphire that can supply high-quality plants with consistent quality from batch to batch.

With the increasing world’s population, the food production must scale-up by 50% to feed the expected 9.9 billion population by 2050 [15,16], which drives the need of establishing new, effective crops. This involves the production of a high number of plants of selected superior traits, such as high nutritional value and stress resistance, leading to increased crop yields [15,17]. However, some species can have low success during germination or be difficult to propagate by cuttings, which makes their propagation by such techniques difficult for large-scale commercial exploitation [16]. These issues push the quest for using alternative production systems, such as in vitro plant tissue culture (PTC) technologies, which may be used to overcome the global crisis faced by agricultural and horticultural production induced by global warming, climate change, desertification, and salinization. Moreover, PTC’s main advantages include the high-scale production of new disease-free plants in a short period of time, regardless of environmental or geographical restrictions, and being able to provide plants throughout the year [18,19]. Despite some halophytes being already commercially produced, such as sea asparagus (*Salicornia* L.) and quinoa (*Chenopodium quinoa* Willd.) [14], the number of profitable commercial farms of these species are still low. Therefore, PTC can support a rapid and continuous production of high number of clones to run a nursery to produce stock plants to supply greenhouse productions [18,19] that help to meet the demand of the worlds’ food supply. Furthermore, it is a valuable commercial approach in the production of nursery stock plants with superior quality and added value and consistent batch to batch quality, thereby increasing their potential economic value [15,16].

PTC protocols were already developed for other non-halophyte *Inula* species, including *I. germanica* L. [20], *I. racemosa* Hook.f. [21], *I. verbascifolia* (Willd.) Hausskn. subsp. *verbascifolia* [22], and *I. royleana* Dc. [23]. However, despite such methods being already established for many halophyte species from 17 different families [24], to our best knowledge, there are no reports on the in vitro multiplication of the golden samphire. Therefore, this work aimed to establish a complete micropropagation process for golden samphire targeting the establishment of plant nurseries that boost the commercial exploitation of this species. For that purpose, a complete plant regeneration protocol was developed for the first time through the enhancement of shoot multiplication from nodal explants, rooting, and acclimatization procedures.

## 2. Results

### 2.1. Explant Surface Sterilization and Establishment

Explant surface disinfection is a crucial step for the successful establishment of in vitro micropropagation protocols. Thus, in this work, nodal explants from golden samphire plants grown in a greenhouse were surface sterilized with 0.5% HgCl_2_ for 5 min before being cultured on Murashige and Skoog (MS) basal medium. This method allowed the disinfection of 94% explants. From these, 32% were brown, and 67% were viable, and the last being divided into nodal segments that were used in the shoot multiplication experiments.

### 2.2. Shoot Multiplication

**Figure 1:**
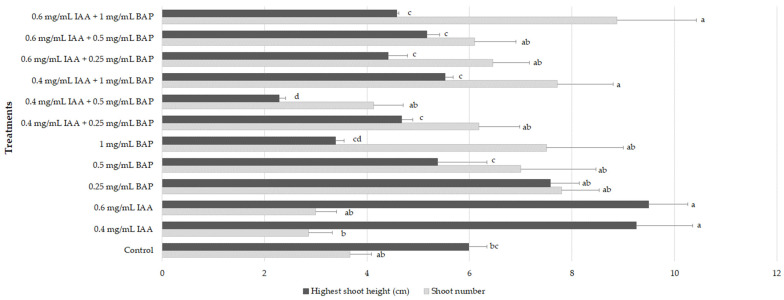
Effect of multiplication treatments on shoot height and number of golden samphire explants cultivated for 6 weeks. Values correspond to mean ± SEM of three independent experiments (n = 30). For each group, columns marked with different letters (a–d) are considered statistically different at *p* < 0.05 (Tukey’s HSD).

Shoot multiplication is a common procedure for plant micropropagation involving the induction of new shoots formation usually by the repeated growth of new axillary branches, being crucial to obtain high number of clones from selected specimens to supply for their subsequent large-scale cultivation [24]. In this work, for golden samphire multiplication, nodal explants were cultivated for 6 weeks in MS medium alone and supplemented with different plant growth regulators (PGRs) only (0.4 mg/L indole-3-acetic acid (IAA), 0.6 mg/L IAA, 0.25 mg/L 6-benzylaminopurine (BAP), and 0.5 mg/L BAP, 1 mg/L BAP) or in combinations (0.4 mg/L IAA + 0.25 mg/L BAP, 0.4 mg/L IAA + 0.5 mg/L BAP, 0.4 mg/L IAA + 1 mg/L BAP, 0.6 mg/L IAA + 0.25 mg/L BAP, 0.6 mg/L IAA + 0.5 mg/L BAP, and 0.6 mg/L IAA + 1 mg/L BAP). Results are presented in Figure 1.

**Figure 2:**
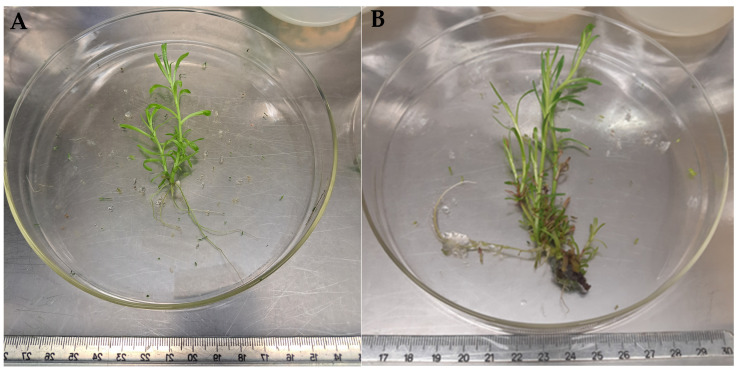
General aspect of 6-week-old golden samphire shoots cultivated in MS basal medium without PGRs (**A**) and MS medium supplemented with 0.25 mg/L BAP (**B**).

When PGRs were applied alone, BAP was more effective in increasing the shoot number at all concentrations tested (7–7.8 shoots/explant) when compared to the control (3.67 shoots/explant). Explants treated with IAA alone showed decreased number of shoots (2.86–3 shoots) (Figure 1; *p* < 0.05). In turn, explants subjected to IAA treatment had increased height (9.26–9.5 cm), whereas those treated with increasing concentrations of BAP exhibited a decrease in shoots height, from 7.58 cm at 0.25 mg/L to 3.39 cm at 1 mg/L (Figure 1). When explants were maintained in MS supplemented with different combinations of BAP and IAA, the combination of IAA (0.4 or 0.6 mg/L) and BAP at 1 mg/L resulted in a significant increase in the formation of new shoots (7.71–8.88). The highest shoot height was achieved with the treatment consisting of 0.4 mg/L IAA + 1 mg/L BAP (5.52 cm) and 0.6 mg/L IAA + 0.5 mg/L BAP (5.17 cm). Overall, the condition that allowed to couple a high formation of new shoots with the highest height was the MS medium supplemented only with 0.25 mg/L BAP (7.8 shoots with 7.58 cm; Figure 2).

Moreover, plants rooted spontaneously on the multiplication media containing cytokinins and auxins (Table 1), particularly those cultivated in control medium and on medium supplemented with 0.4 and 0.6 mg/L IAA and with 0.25 and 1 mg/L BAP alone where 100% of explants developed roots. The other conditions presented lower number of rooted explants (Table 1).

Explants cultured with different combinations of BAP and IAA as well as with 0.5 and 1 mg/L BAP alone induced the growth of dark-green callus (75–100% of callogenesis; Table 1). Furthermore, none of the conditions promoted the occurrence of hyperhydricity or shoot tip necrosis.

### 2.3. Rooting

**Figure 3:**
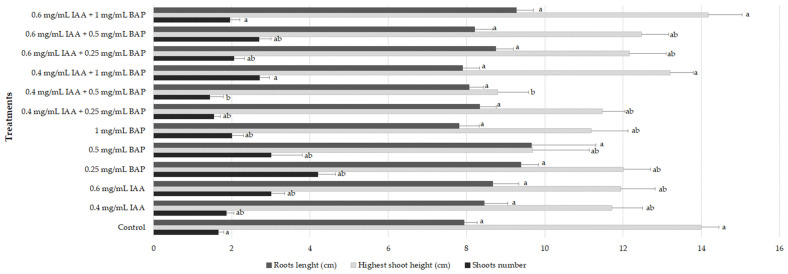
Root length, shoot height, and number of explants obtained in different multiplication media. Values correspond to mean ± SEM of three independent experiments (n = 30). For each group, columns marked with different letters (a–b) are considered statistically different at *p* < 0.05 (Tukey’s HSD).

The development of healthy and well-developed root systems by in vitro-produced shoots is essential for their successful establishment in the soil and survival in ex vitro acclimatization [16]. In this work, in order to root the new shoots obtained from the different multiplication treatments, they were cultivated in MS medium free of PGRs for 6 weeks. All shoots were able to develop roots (100% rooting), and the effect of the multiplication conditions on the in vitro rooting was assessed by measuring root length, shoot number, and height. Results are depicted in Figure 3.

**Figure 4:**
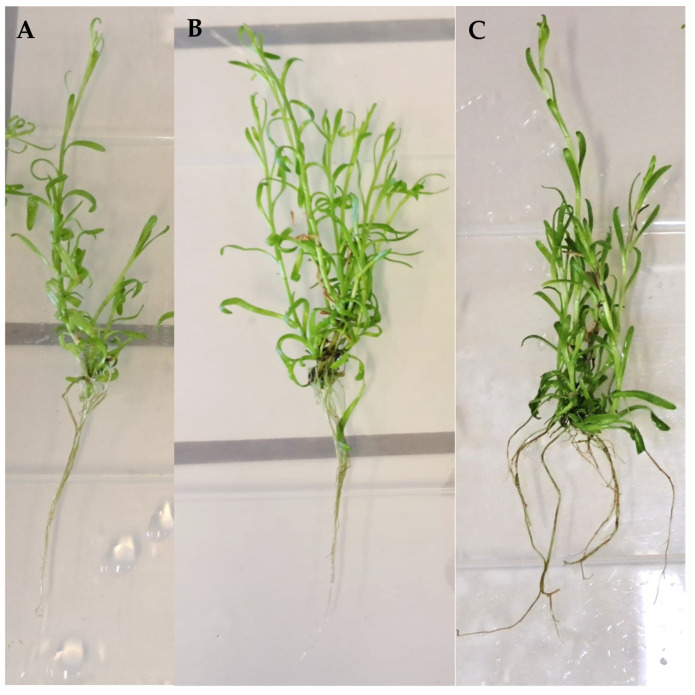
General aspect of golden samphire plantlets after 6 weeks of in vitro rooting in MS basal medium derived from multiplication media: MS medium alone (**A**) and MS medium supplemented with 0.25 mg/L BAP (**B**) and 0.6 mg/L IAA + 1 mg/L BAP (**C**).

Generally, the multiplication conditions did not affect the rooting ability of the plantlets since no significant differences were found regarding the root length amongst the plants derived from the different multiplication treatments and when compared to plants cultivated in MS basal medium without PGRs (7.8–9.7 cm/plantlet, *p* < 0.05) (Figure 3). Regarding the formation of new shoots, at the end of the rooting phase, plantlets maintained in medium containing 0.25 mg/L BAP showed the highest number (4.2 shoots/plantlet), and plantlets derived from 0.6 mg/L IAA + 1 mg/L BAP showed the highest shoot height (14.2 cm), similar to those cultivated in control conditions (14.0 cm) (Figure 3 and Figure 4).

During plant in vitro culture, shoot-tip necrosis (STN) can be a major problem in the successful propagation of many species. The symptoms include browning and loss of new buds and young leaves [25]. In this study, by the end of the rooting phase, several plants presented signs of STN, especially the ones derived from MS basal medium free of PGRs (58%) (Table 2). Moreover, the presence of IAA during multiplication step seems to increase the occurrence of STN, except when it was applied alone at the lowest concentration (0.4 mg/L). BAP seems to have a low effect on STN development (Table 2).

### 2.4. Acclimatization

The ultimate success of the in vitro propagation process is the effective establishment of plants in the soil and to the ex-vitro conditions; therefore, the acclimatization to ex vitro conditions must be as gradual as possible to increase plant survival [16,17]. Therefore, at the end of the rooting phase, rooted plantlets were transferred to plastic trays containing peat + vermiculite (3:1) and progressively acclimatized to culture room conditions using lidded plastic boxes that were gradually opened from 1 h/day until staying completely open after 6 weeks. The percentage of plant survival and plant height were then determined (Table 3).

**Figure 5:**
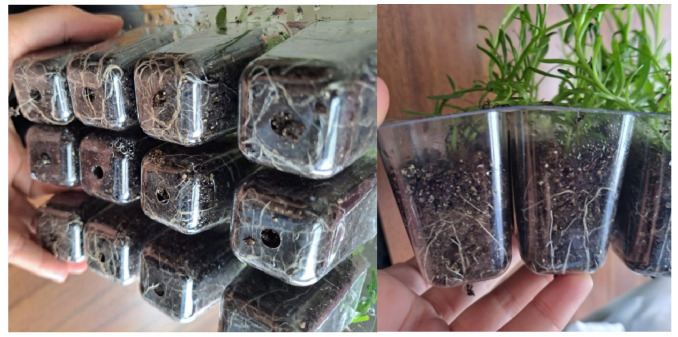
General aspect of root system of golden samphire plants after 6 weeks of acclimatization.

Only 9.8% of the plants without any treatment (control) survived the acclimatization process, whereas the survival of plants that were sprayed with a solution of 17.5% paraffin prior to acclimatization increased to 83.3% (Table 3). Moreover, plants treated with paraffin solution achieved a height of 18.3 cm, showing a growth of more than 4 cm in 6 weeks compared to plantlets grown under control conditions at the end of rooting phase. The root system was well-developed after 6 weeks of acclimatization in plants treated with paraffin solution (Figure 5).

## 3. Discussion

The golden samphire is an edible halophyte species rich in iodine, proteins, carotenoids, water-soluble vitamins (B1 and B6) and important minerals (sodium, potassium, and iron) and with antioxidant and antibacterial properties [10,11,12,13,14]. Besides their nutritional and medicinal properties, this plant has high-organoleptic qualities that make it highly attractive for human consumption [14]. The most common techniques used for the cultivation of commercial crops are based on sexual and vegetative propagation. However, such methods are frequently ineffective for species that have low rates of seed germination or that are difficult to multiply by vegetative propagation methods, which makes their propagation by such techniques difficult for large-scale commercial exploitation [16]. Therefore, in vitro culture methods may be advantageous to supply the high-scale commercial production of these species, such as golden samphire, being actually essential to develop a continuous and rapid large-scale nursery method that allows provision of a high number of healthy plants devoid of seasonal and geographic constraints [16,18]. In this context, this work aimed at developing a micropropagation protocol for the golden samphire to be used as a nursery method for its potential commercial production.

Microbial contamination is one of major restrictions in micropropagation procedures, causing difficulties in culture initiation. Thus, surface sterilization of explants is a crucial step. In this regard, HgCl_2_ was used in this study; however, its use is associated with high toxicity in explants and consequent dead [26], which is possibly related to the high browning of the explants that we observed in this study. To increase viability of explants, other less toxic sterilization solutions must be used, namely, ethanol, sodium hypochlorite, or calcium hypochlorite, as reported for the in vitro culture of other halophytes species [24]. 

Data regarding the micropropagation of *Inula* species is scarce, with only five studies involving four species, namely, *I. racemosa*, *I. verbascifolia* subsp. *verbascifolia*, *I. germanica*, and *I. royleana* [20,21,22,23]. Most similar to our work was the regeneration protocol developed for *I. racemosa* nodal segments obtained from in vitro germinated seeds and cultivated in MS medium supplemented with 0.25 mg/L BAP alone that had the maximum number of shoots (20.7 shoots/explants) [21]. However, the multiplication rate was significantly higher that the value obtained in this work (7.8 shoots/explant), for the same condition, most probably due to the different species used [21]. Cytokinins, such as BAP, have a key role in plant development, i.e., stimulating cell differentiation and promoting the growth of side shoots and apical dominance and expansion [27], which explains the higher shoot number obtained by explants treated with BAP alone. Actually, BAP has already shown to increase shoot multiplication of several medicinal plant species, especially those belonging to the Asteracae family [28], which complies with the results of this study. In turn, the highest length of shoots treated with IAA alone is may be related to its reported capacity to promote cell division and shoot elongation, which is known to contribute to the increase in shoot height rather than shoot number [29].

Moreover, the number of shoots and their height generally have a negative correlation, i.e., shoots from explants with a higher shoot number were generally shorter than those from explants with lesser new shoots formation. Auxins and cytokinins may be combined to improve plant growth and differentiation, but their simultaneous application may lead to antagonistic effects, and therefore, shoot proliferation and elongation can be reduced when compared to their application alone. For instance, other authors also evaluated the effect of the application of different cytokinin and auxin combinations on the in vitro multiplication of *Inula* species, namely, Stojakowska and Malarz [23] who developed a method to micropropagate *I. royleana* from cotyledonary node explants by cultivation in MS medium supplemented with 0.1 µM NAA and 5.0 µM KIN, with the regeneration of 3.4 and 5.1 shoots/explant, respectively. Furthermore, seedling explants from *I. germanica* grown in MS medium supplemented with 1 mg/L BAP and 0.1 mg/L NAA resulted in the formation of 6.3 shoots/explant [20]. However, these reports presented lower shoot multiplication rates that the ones using cultivation media supplemented with BAP alone.

A complete and effective micropropagation protocol involves the optimization of the optimum conditions for the growth of new shoots and in vitro root initiation. Moreover, shoots with healthy and well-developed root systems are more likely to establish themselves in the soil and present higher survival rates during the ex-vitro acclimatization [16]. In this work, all the golden samphire shoots have successfully rooted in MS basal medium similar to other Inula species that have been reported to develop roots on hormone-free media, namely, *I. verbascifolia* subsp. *verbascifolia* (67%), and *I. royleana* (70%) [22,23]. However, the spontaneous rooting in media containing cytokinins, such as BAP, as observed in this work, was also reported for *I. verbascifolia* subsp. *verbascifolia* [22]. This suggests that the usual three-stage in vitro procedure used here might not be necessary for the in vitro propagation of the golden samphire, but a two-stage procedure can be rather applied, making the micropropagation process faster and more effective and allowing to obtain acclimatized plants in a shorter period of time.

By the end of the rooting phase, more than half of the plants derived from MS basal medium showed signs of shoot-tip necrosis as well as most of the plants that were multiplied in IAA-containing media. Actually, there are several factors that commonly contribute to the occurrence of STN, for example, nutrient deficiency or imbalance, presence or absence of plant growth regulators, high relative humidity, adequate supply of calcium (Ca^2+^), and high ethylene production as well as the timing of the measurements and the subculture interval [25]. Moreover, reducing the subculture interval, reducing concentration of basal media strength, adjusting sucrose levels, and/or increasing flask ventilation or using different PGRs can be used as approaches to reduce the signs of STN in the in vitro-produced *Inula* plants.

The acclimatization stage is critical to in vitro-produced plantlets since they have low cuticular waxes and low- or non-active stomatal systems that lead to rapid desiccation of in vitro plantlets, which may result in low survival rates during ex-vitro acclimatization [30,31], such as those observed in this work for plants without any treatment (control). To overcome these problems, a proper acclimatization approach and selection of the acclimatization medium should be improved, which may include using antitranspirants, such as abscisic acid (ABA), or increasing the photosynthetic rate by increasing the CO_2_ level [18,30,31]. Furthermore, an in vitro hardening step before acclimatization can contribute to increase in the survival percentage at the acclimatization stage, for example, by increasing or reducing sucrose concentration in the media, using desiccants, or increasing air exchanges [31,32]. In this context, a simple spraying of a paraffin solution prior to acclimatization was enough to increase the plant survival by 8.5 times. Paraffin forms a waxy layer on the leaves surface, working as an antitranspirant by decreasing water loss and enhancing the water status of plant, and decreasing wilting and leaf abscission [32]. Overall, a complete micropropagation protocol was successfully developed for golden samphire, indicating its potential establishment as a nursery method to boost the commercial exploitation of this species.

## 4. Materials and Methods

### 4.1. Plant Material and Explant Preparation and Disinfection

Seeds of *I. crithmoides* were collected in September 2020 in Olhão (37°01′12.0″ N 7°53′04.8″ W) and were germinated using irrigation with freshwater in May 2021. Once germinated (10–15 days), they were transferred to brackish water irrigation (20.1 mS/cm). In March 2022, aerial parts (leaves and stems) were collected from greenhouse-grown plants, washed under running tap water, immersed in tap water with commercial soap for 15 min, and repeatedly rinsed with distilled water. The leaves were then removed and discarded, and the stems were divided into nodal segments of 2 cm and used as explants that were surface sterilized by immersion in 0.5% mercury (II) chloride (HgCl_2_) for 5 min and rinsed 3 times with sterile distilled water.

### 4.2. Establishment

The explants were placed in individual glass tubes (20 × 2.0 cm) containing 15 mL of MS basal medium (Murashige and Skoog, 1962) free of plant growth regulators (PGRs), supplemented with 2% sucrose and 0.8% agar, and maintained under the temperature of 25 ± 2 °C, with a 16/8 h light/dark photoperiod provided by LED light (2700 Kelvin) at 3000 lux of light intensity. Explants were maintained until enough shoots were available to establish the multiplication experiments. All media were adjusted to pH 5.8 prior to autoclaving at 121 °C for 20 min.

### 4.3. Multiplication and Rooting

Shoots generated from disinfected explants were distributed into different groups for sub-cultivation, including (1) control plants grown with MS medium free of PGRs, and 11 treatments with MS medium supplemented with different combinations of growth regulators, namely, (2) 0.4 mg/L IAA, (3) 0.6 mg/L IAA, (4) 0.25 mg/L BAP, (5) 0.5 mg/L BAP, (6) 1 mg/L BAP, (7) 0.4 mg/L IAA + 0.25 mg/L BAP, (8) 0.4 mg/L IAA + 0.5 mg/L BAP, (9) 0.4 mg/L IAA + 1 mg/L BAP, (10) 0.6 mg/L IAA + 0.25 mg/L BAP, (11) 0.6 mg/L IAA + 0.5 mg/L BAP, and (12) 0.6 mg/L IAA + 1 mg/L BAP. Cultures were maintained for 6 weeks for multiplication, and the generated shoots from the different conditions were separated and transferred to MS basal medium free of PGRs for rooting for 6 weeks. The shoots were counted, and the height of the highest shoot was measured at the beginning of the multiplication phase and at the end of the rooting phase. All media were adjusted to pH 5.8 prior to autoclaving at 121 °C for 20 min.

### 4.4. Acclimatization

For the ex-vitro acclimatization, the two groups of rooted plantlets, (1) control—no treatment and (2) plants sprayed with a solution of 17.5% paraffin, were transferred to plastic cuvettes with peat + vermiculite (3:1, *v*/*v*) as the substrate, placed in plastic boxes covered by a transparent lidded plastic box, and then placed indoors at room temperature (RT, 25 ± 2 °C) with high humidity (90–100%) and natural light (2500–3000 lux) for 7–10 days. Plants were gradually adapted to culture room conditions (55–60% humidity, 2500–3000 lux, and 25 ± 2 °C) by gradually opening the lid from 1 h/day until staying completely open at 6 weeks. Then, plant survival (%) and plant height (cm) were measured. To prevent fungal contaminations, an antifungal commercial solution (benzothiazolinone 0.4%, *v*/*v*) was applied weekly.

### 4.5. Statistical Analyses

Results are expressed as mean ± standard error of the mean (SEM), and experiments were performed at least in triplicates. Significant differences were evaluated using ANOVA and Tukey’s honestly significant difference (HSD) test (*p* < 0.05). Statistical analyses were conducted using the XLSTAT statistical package for Microsoft Excel (version 2013, Microsoft Corporation, Redmond, WA, USA).

## 5. Conclusions

The in vitro establishment, multiplication, rooting, and acclimatization protocols for the edible and medicinal halophyte golden samphire were successfully developed from shootless nodal segments, and plantlets were derived from new vegetative shoots devoid of morphological changes that were continuously multiplied. Moreover, plenty of well-rooted plants can be obtained in only two stages, i.e., initiation and multiplication, allowing to obtain plants in a shorter period of time. The ex-vitro acclimatization process was highly improved by applying a paraffin solution on the surface of leaves. Overall, the micropropagation of the golden samphire showed to be a promising way for a rapid and improved multiplication of this species, which can be used as a nursery to supply a high-scale commercial production, contributing to developing it as an alternative food and medicinal crop.

## List of Abbreviations

ABAAbscisic acidBAP6-BenzylaminopurineIAAIndole-3-acetic acidKINKinetinMSMurashige and SkoogNAA1-Naphthaleneacetic acidPGRsPlant growth regulatorsPTCPlant tissue cultureSTNShoot-tip necrosis

## Figures and Tables

**Table 1:** Percentage of rooting and callus formation of golden samphire explants subjected to different shoot multiplication media after 6 weeks of cultivation.

Treatments		Rooting (%)	Callus (%)
IAA (mg/L)	BAP (mg/L)		
0	0	100	0
0.4	0	100	0
0.6	0	100	0
0	0.25	100	0
0	0.5	50	75
0	1	100	100
0.4	0.25	41	100
0.4	0.5	13	100
0.4	1	76	100
0.6	0.25	64	100
0.6	0.5	70	100
0.6	1	88	100

**Table 2:** Percentage of shoot-tip necrosis of golden samphire explants subjected to different shoot multiplication media after 6 weeks of rooting.

Treatments		Shoot-Tip Necrosis (%)
IAA (mg/L)	BAP (mg/L)	
0	0	58
0.4	0	0
0.6	0	30
0	0.25	6
0	0.5	0
0	1	0
0.4	0.25	18
0.4	0.5	22
0.4	1	41
0.6	0.25	5
0.6	0.5	19
0.6	1	33

**Table 3:** Survival (%) and plant height (cm) of golden samphire plants after 6 weeks of acclimatization.

Treatment	Survival (%)	Plant Height (cm)
Control	9.80	-
Paraffin	83.3	18.3 ± 0.84

## Data Availability

The dataset is available upon request from the corresponding author.
